# Application of MicroRNA in Diagnosis and Treatment of Ovarian Cancer

**DOI:** 10.1155/2014/232817

**Published:** 2014-04-15

**Authors:** Kouji Banno, Megumi Yanokura, Miho Iida, Masataka Adachi, Kanako Nakamura, Yuya Nogami, Kiyoko Umene, Kenta Masuda, Iori Kisu, Hiroyuki Nomura, Fumio Kataoka, Eiichiro Tominaga, Daisuke Aoki

**Affiliations:** Department of Obstetrics and Gynecology, School of Medicine, Keio University, Shinanomachi 35, Shinjuku-ku, Tokyo 160-8582, Japan

## Abstract

Ovarian cancer has a poor prognosis because early detection is difficult and recurrent ovarian cancer is usually drug-resistant. The morbidity and mortality of ovarian cancer are high worldwide and new methods of diagnosis and therapy are needed. MicroRNAs (miRNAs) are posttranscriptional regulators of gene expression that are involved in carcinogenesis, metastasis, and invasion. Thus, miRNAs are likely to be useful as diagnostic and prognostic biomarkers and for cancer therapy. Many miRNAs have altered expression in ovarian cancer compared to normal ovarian tissues and these changes may be useful for diagnosis and treatment. For example, deficiencies of enzymes including Dicer and Drosha that are required for miRNA biogenesis may be adverse prognostic factors; miRNAs such as miR-214 and miR-31, which are involved in drug resistance, and the miR-200 family, which is implicated in metastasis, may serve as biomarkers; and transfection of downregulated miRNAs and inhibition of upregulated miRNAs may be effective for treatment of ovarian cancer. Chemotherapy targeting epigenetic mechanisms associated with miRNAs may also be effective to reverse gene silencing.

## 1. Introduction

Ovarian cancer is the eighth most common female cancer worldwide and ranks seventh in mortality. About 220,000 women are diagnosed with ovarian cancer each year and the disease causes about 140,000 deaths annually [1]. In Japan, the incidence and mortality of ovarian cancer have increased over the past 10 years [2, 3]. The 5-year survival rate for patients with advanced ovarian cancer is only 30%, despite the development of chemotherapy with platinum-based drugs and taxanes [4]. The high mortality is associated with difficulties in early detection because ovarian cancer rarely causes subjective symptoms and safe and minimally invasive procedures for early detection have not been established. Consequently, 40% to 50% of cases are detected in advanced stages III and IV. Another cause of the high mortality is resistance to chemotherapy. Ovarian cancer is highly responsive to initial anticancer treatment, but about half of the advanced cases recur within two years and have a decreased response to chemotherapy, resulting in a poor prognosis [5]. For these reasons, there is an urgent need to develop new therapies, find clinically useful biomarkers, and identify new targets for treatment of ovarian cancer.

Many studies of ovarian cancer have focused on protein-coding genes. However, RNA molecules transcribed from noncoding genes also have biological functions. These noncoding RNAs include microRNAs (miRNAs) that cleave a target mRNA and repress translation of proteins, and some miRNAs show site- and stage-specific differences in expression in ovarian cancers. Many recent studies have shown that miRNAs are involved in suppression or progression of ovarian cancer. Therefore, miRNAs may be useful as diagnostic and prognostic biomarkers and also for therapy. Epigenetic therapy related to miRNAs may be particularly effective for resensitization of ovarian cancer cells to chemotherapy after development of resistance and recurrence. In this paper, we describe the possible use of miRNAs in diagnosis and treatment of ovarian cancer.

## 2. miRNAs Implicated in Ovarian Function

Ovarian function, particularly follicular development, is controlled by hormones such as gonadotropins, including follicle-stimulating hormone (FSH) and luteinizing hormone (LH). The ovary itself also produces sex hormones such as progesterone and estrogen, as well as cytokines of the transforming growth factor beta (TGF-*β*) superfamily [6]. A recent study suggested cross talk between the signals of local ovarian factors and endocrine system hormones including gonadotropins [7]. Thus, ovarian function is controlled by complex molecular signaling that maintains normal follicular development and atresia, in which protein expression is regulated quantitatively and temporally. Failure of the regulatory mechanisms is likely to lead to various diseases, including infertility.

Follicular cells are roughly classified into theca cells, granulosa cells, and oocytes. Granulosa cells proliferate in a FSH-dependent manner during follicular maturation and are involved in estrogen synthesis. Mase et al. found that many miRNAs in the let-7 family are expressed in human ovarian granulosa KGN cells, which maintain expression of FSH receptors. Genes targeted by the let-7 family include those involved in follicular maturation and atresia, suggesting involvement of miRNAs in these phenomena [8]. Murchison et al. produced Dicer 1 knockout mice with oocyte-specific deletion of Dicer, an important enzyme for miRNA biogenesis. In these mice, functional expression of miRNAs was completely deleted. Dicer deletion had no effect on early folliculogenesis but arrested the first meiotic division in oocytes associated with spindle and chromosomal aggregation hypoplasia. More than 2,000 mRNAs had significantly changed expression associated with these abnormalities. During oocyte maturation, including meioses, gene transcription was completely repressed and only mRNAs inherited before maturation remained in cells. Expression of many mRNAs was affected in Dicer-deleted oocytes, suggesting direct or indirect posttranscriptional regulation by miRNAs [9].

## 3. Changes in miRNA Expression in Ovarian Cancer 

Recent studies have identified many oncogenic miRNAs (oncomiRs) and tumor suppressor miRNAs (tumor suppressor miRs) (Table 1) [10–16]. Iorio et al. found several miRNAs with altered expression in ovarian cancer tissues compared with normal tissues, with miR-199a, miR-200a, miR-200b, and miR-200c having significantly increased expression and miR-140, miR-145, and miR125b1 showing markedly decreased expression in the cancer tissues. miR-140 is located at 6q22, a common defective chromosomal site in ovarian tumors, and this miRNA is thought to target genes associated with invasion, including matrix metalloproteinase 13, fibroblast growth factor 2, and angiogenic VEGFA [10]. Bracken et al. showed that miR-429, miR-200a, and miR-200b are regulated by ZEB1 and SIP1, which are inhibitors of the epithelial-mesenchymal transition (EMT), and that miR-200a and miR-200b negatively regulate expression of ZEB1 and SIP1, providing a negative feedback loop [11].

With regard to miRNA processing of mRNA, an interesting study of the relationship of ovarian cancer with miRNAs by Merritt et al. [12] showed that the mRNA levels of Dicer and Ribonuclease 3 (Drosha) decreased in 60% and 51% of tissue samples from 111 patients with invasive epithelial ovarian cancer. Downregulation of Dicer was significantly related to tumor stage progression and downregulation of Drosha was significantly related to a suboptimal residual tumor size >1 cm after cytoreductive surgery. Conversely, patients with high levels of Dicer and Drosha in cancer tissues had significantly prolonged median survival times. Cells with downregulation of Dicer and Drosha are likely to have lower levels of mature miRNAs, which suggests that certain miRNAs are involved in progression of ovarian cancer [12].

## 4. miRNAs Associated with Drug Resistance

A total of 27 miRNAs have been associated with responsiveness to chemotherapy [13]. Yang et al. found that miR-214, which targets PTEN, is frequently expressed in ovarian cancer tissues and that let-7i, which enhances resensitization to platinum resistance, is expressed less in the same tissues [14]. Mitamura et al. showed that control of MET expression by miR-31 is involved in drug-resistance mechanism in paclitaxel-resistant ovarian cancer cells [15]. Aqeilan et al. found that miR-15 and miR-16 cause cellular resistance to many drugs through targeting the BCL2 gene [16]. Leskelä et al. showed that the miR-200 family (miR-141, miR-200a, miR-200b, miR-200c, and miR-42) is implicated in the response to paclitaxel treatment and progression-free survival via *β* tubulin III regulation. In particular, miR-200c is significantly associated with recurrence of ovarian cancer and miR-429 is associated with progression-free and overall survival rates [17].

Key drugs against ovarian cancer are taxanes and cisplatin. Boyerinas et al. found that let-7g and let-7a are involved in drug resistance [18]. Let-7g suppresses IMP-1, which is involved in multidrug resistance and increased sensitivity to taxanes. The expression level of let-7a is a potential marker for choosing chemotherapeutic agents, since patients with extremely low let-7a expression are responsive to platinum-based drugs and paclitaxel, whereas those with high levels of let-7a had increased survival only in monotherapy with a platinum-based drug [19]. Nagaraja et al. and Peng et al. showed that miR-100, a tumor suppressor miRNA, increased sensitivity to everolimus, an anticancer drug [20, 21]. miR100 is also an independent predictor of overall survival in patients with ovarian cancer. Hong et al. showed that miR-376c suppresses signaling of Nodal/activin receptor-like kinase 7 (ALK7), which is involved in drug sensitivity, and decreases the effects of cisplatin and carboplatin [13]. Fu et al. found that miR-93 targets integrin and enhances tumor growth, angiogenesis, and the resistance for cisplatin [22].

## 5. Utility of miRNAs in Diagnosis of Ovarian Cancer

Many miRNAs have altered expression levels in ovarian cancer compared to normal tissues. In addition, changes in miRNA levels are dependent on and related to the ovarian cancer tissue type, stage, histological type, prognosis, and drug resistance (Table 2) [8, 10, 23–30]. These findings suggest the possibility of early diagnosis of ovarian cancer using miRNAs. In the miR-200 family, Boyerinas et al. showed that miR-200a and miR-200c are expressed in serous adenocarcinoma, clear cell adenocarcinoma, and endometrioid adenocarcinoma, and miR-200b and miR-141 occur in endometrioid adenocarcinoma and mucinous adenocarcinoma [18]. Toloubeydokhti et al. found decreased expression of miR-212 in serous cystadenoma [31]. Target genes of miR-212 include those with overexpression in this histological type of ovarian cancer and mutated genes in hereditary ovarian cancer. Therefore, miR-212 may be a marker for differentiating ovarian cancer. Downregulation of miR-31, a tumor suppressor miRNA, has been shown in serous adenocarcinoma, and miR-31 suppresses expression of cell cycle regulatory factors via p53 [32]. Expression of miR-373 is variable in undifferentiated carcinoma [33], but the target genes and function of this miRNA are unknown. Overexpression of miR-21 in clear cell carcinoma has been shown to cause downregulation of PTEN [34].

## 6. Utility of miRNAs in Treatment of Ovarian Cancer

Treatment options for ovarian cancer include supplementation of miRNAs that are downregulated in cancer tissue for recovery of function and inhibition of the function of upregulated miRNAs by administration of complementary nucleic acids. Garzon et al. showed that the effects of upregulated oncomiRs could be suppressed using an antagomir, an oligonucleotide complementary to the miRNA administered as an antisense oligonucleotide or LNA [35]. Lu et al. developed an anti-miRNA antisense oligodeoxyribonucleotide (MTG-AMO) for suppression of many miRNAs, including miR-21, and showed that this was effective in cancer with concurrent multiple mRNA abnormalities [36]. Dai et al. established a therapy for ovarian cancer based on targeted delivery of miR-29a to cancer tissues for the purpose of reexpressing PTEN, a tumor suppressor. The potential antitumor effect of a miR-29a-transfected chimera was apparently based on expression of downstream molecules and apoptosis of ovarian cancer cells [37].

The association of miRNAs with peritoneal metastasis, the major cause of death in patients with ovarian cancer, has also been studied. Ohyagi-Hara et al. found that integrin *α*5, a fibronectin receptor, increased the adhesion of cancer cells and induced metastasis and focused on the inverse correlation of integrin *α*5 and miR-92a levels. Transfection of ovarian cancer cells with miR-92a reduced expression of integrin *α*5 and suppressed peritoneal metastasis [38]. Cittelly et al. found that recovery of the level of miR-200c, which is known to increase sensitivity to platinum-based anticancer drugs, by transfection suppressed carcinogenesis and decreased the number of cancer cells. Recovery of miR-200c in combination with paclitaxel also decreased the cancer cells in established tumors. These results suggest that recovery of miR-200c immediately before highly cytotoxic chemotherapy improves the treatment response or reduces the effective dose of the anticancer drugs [39]. These outcomes show that miRNA transfection has an antitumor effect. Transfected miRNAs are synthetic nucleic acids that require specific modes of administration [40–42]. These approaches include intravenous administration of a complex with atelocollagen, nanoparticles with cell-specific targeting, and conjugation with RVG peptide for crossing the blood-brain barrier. Gene therapy for introduction of miRNAs may also be useful if safety can be confirmed.

Epigenetic therapy has attracted attention as an alternative to classical approaches such as miRNA transfection. Acquisition of drug resistance reduces the survival rate in cancer and many cases of ovarian cancer are resistant to platinum-based anticancer drugs. This resistance is associated with miRNAs and various drug-resistance genes induced by methylation and signaling gene silencing. However, epigenetic changes are reversible, in contrast to gene mutations, and there is a potential to reverse gene silencing using DNA methyltransferase (DNMT) inhibitors, which are drugs that prevent hypermethylation by irreversibly binding to the active site of DNMT [43]. These drugs are effective as monotherapy for hematologic malignancy [44, 45], but not in solid cancer. However, effects on solid cancer are likely to be found in combination with other drugs. In ovarian cancer cells, DNMT inhibitors induce hypomethylation and reverse resistance to platinum-based anticancer drugs. Phases I and II clinical trials of decitabine, a DNMT inhibitor, are ongoing in ovarian cancer [46, 47]. Matei et al. found that decitabine in combination with carboplatin restored the expression of silenced tumor suppressor genes and may contribute to resensitization of platinum-resistant endometrial cancer. A phase I clinical trial of decitabine has shown that combined administration with carboplatin is safe and decreases methylation of multiple genes* in vivo* [48].

Malignant tumors, including ovarian cancer, include cancer initiation cells and cancer stem cells, which are referred to as cancer progenitor cells and are involved in development of drug resistance [49–51]. Chemotherapy targeting mitosis cannot eliminate all cancer stem cells during cell cycle arrest or low activity conditions, and residual cells promote regrowth of the tumor. Epigenetic therapy stabilizes differentiation and may target undifferentiated cancer stem cells. Thus, targeting of epigenetic mechanisms is likely to improve outcomes in ovarian cancer.

## 7. Conclusion

miRNAs have attracted significant interest, but the history of this field is relatively short and many issues remain to be resolved. Clinical studies of miRNAs have just started, but functional genomic analyses have produced results that may lead to clinical applications in the near future. Early diagnosis of ovarian cancer is important to improve treatment outcomes, and profiling using miRNA arrays may contribute to the detection of tissue type, stage, and prognosis. Induction of apoptosis of cancer cells using miRNAs may be a basic treatment strategy for reduction of metastasis, including peritoneal metastasis, and decreasing resistance to platinum-based anticancer drugs. Recovery of tumor suppression effects may be possible by transfection of miRNAs downregulated in cancer tissues or by suppression of upregulated miRNAs. miRNA expression may also be modified by targeting epigenetic mechanisms such as through reversal of hypermethylation. These potential treatment approaches will require further basic studies to facilitate drug discovery.

## Figures and Tables

**Table 1:** Changes in miRNA expression in ovarian cancer.

Upregulated (oncomiRs)	Downregulated (tumor suppressor miRs)
miR-199a	miR-140
miR-200a	miR-145
miR-200b	Let-7i
miR-200c	miR-15
miR-429	miR-16
	miR-373
	miR-520c
	miR-125b1

**Table 2:** Histological types and miRNA expression in ovarian cancer.

Tissue type	Upregulation	Downregulation
Serous adenocarcinoma	miR-7 miR-200a/c	miR-148b
	miR-22 miR-302b	miR-211
	miR-373 miR-34c-5p	miR-31
	miR-449a miR-146b-5p	

Endometrioid adenocarcinoma	miR-9 miR-183 miR-205	miR-22 miR-222 miR-324-3p
	miR-96 miR-196a miR-212	miR-101 miR-299-5p miR-325
	miR-182 miR-196bmiR-375	miR-194 miR-302b miR-373
	miR-141 miR-200a/b/c	

Clear cell adenocarcinoma	miR-29b miR-200a/c	miR-20a
	miR-30a miR-486-5p	
	miR-30e	

Mucinous adenocarcinoma	miR-141 miR-200b	

Undifferentiated carcinoma		miR-9 miR-18
